# Validity and reliability of the Malay Iowa Infant Feeding Attitude Scale among mothers with infants in Malaysia: A validation study

**DOI:** 10.51866/oa.518

**Published:** 2025-11-29

**Authors:** Nurjasmine Aida Jamani, Hazwani Hanum Hashim, Karimah Hanim Abd Aziz, Nor Azam Kamaruzaman

**Affiliations:** 1 MD, M.Med (Fam Med), Department of Family Medicine, Faculty of Medicine and Health Sciences, University Malaysia Sabah, Jalan UMS, Kota Kinabalu, Sabah, Malaysia. Email: hazwanihanum@ums.edu.my; 2 MD, M.Med (Fam Med), IBCLC, Department of Family Medicine, Kulliyyah of Medicine, International Islamic University Malaysia, Jalan Sultan Ahmad Shah, Bandar Indera Mahkota, Kuantan, Pahang, Malaysia.; 3 MD, MPH, DrPH, Department of Community Medicine, Kulliyyah of Medicine, International Islamic University Malaysia, Jalan Sultan Ahmad Shah, Bandar Indera Mahkota,Kuantan, Pahang, Malaysia.; 4 MD, M.Med (Fam Med), Department of Family Medicine, Kulliyyah of Medicine, International Islamic University Malaysia, Jalan Sultan Ahmad Shah, Bandar Indera Mahkota, Kuantan, Pahang, Malaysia.

**Keywords:** Infant nutrition, Breastfeeding, Language, Psychometrics, Maternal behaviour

## Abstract

**Introduction:**

The Iowa Infant Feeding Attitude Scale (IIFAS) has been developed to assess maternal attitudes towards infant feeding choices to predict breastfeeding intention. Validity and reliability need to be established considering cultural, language and environmental differences, especially in the Malay context due to currently unavailable Malay version. This study aimed to translate the IIFAS into Malay (IIFAS-M) and determine its validity and reliability.

**Methods:**

A validation study using a digital survey was conducted. The final translated version was distributed to mothers with infants using the digital form. Principal component analysis was performed using SPSS version 27.

**Results:**

A total of 272 mothers with a mean age of 30.1 (SD±4.2) years participated in the study. A new scoring was given to the IIFAS-M, representing positive attitudes towards breastfeeding and neutral and positive attitudes towards formula feeding. The principal component analysis showed that all items had a factor loading of more than 0.4. The Cronbach’s alpha value was 0.657, and the P-value for the test-retest Pearson correlation was <0.001.

**Conclusion:**

The IIFAS-M is a validated and reliable tool for assessing maternal attitudes towards infant feeding among Malay speakers.

## Introduction

The growth, life-long health and well-being of infants are mainly affected by the nutrition and nurturing of their caretakers. Breastfeeding is the optimum way to provide the best nutrition and has been recommended in infants for the first 6 months of life. After the age of 6 months, complementary feeding is introduced. Nonetheless, milk is still an important source of nutrition until the child reaches the age of 2 years.^1^

Currently, parents have the choice of feeding their infants either with breastmilk, formula milk or both. With these infant feeding options available, mothers’ choices are influenced by various factors. The study by Abdul Hamid et al.^2^ in Selangor, Malaysia, found that women with positive attitudes towards breastfeeding, along with supportive partners and mothers, were significantly more likely to intend to breastfeed their infants.^2^ The Malaysia National Health and Morbidity Survey 2016 showed that 93.2% of mothers’ own decisions influenced their feeding method.^3^ Therefore, attitudes towards infant feeding play an important role in determining feeding choices. However, scholars remain uncertain about maternal attitudes towards feeding across different contexts. In their study, Ara et al. found that only 50% of mothers managed child feeding effectively, while 83.5% possessed a satisfactory comprehension of the practice. Despite many opting for formula feeding during the initial 6 months, 72.5% of women exhibited favourable opinions towards breastfeeding.^4^ Consequently, maternal attitudes directly influence the nutritional outcomes of children. Children whose mothers exhibited negative feeding attitudes were found to have lower BMI.^5^ Conversely, mothers with positive feeding attitudes were more inclined to provide appropriate complementary foods, hence enhancing their children’s nutrition.^6^ Thus, educational interventions are necessary to bridge the knowledge-practice gap and ensure that positive attitudes lead to improved dietary behaviours.^4^

As attitudes are one of the important factors related to infant feeding choices, multiple tools have been developed to assess attitudes towards infant feeding. Casal et al. presented an overview of instruments used to evaluate breastfeeding-related knowledge and attitudes, reviewing six tools that measure attitudes towards infant feeding.^7^ The study outlined the number of items, reliability and availability of tools related to attitudes towards infant feeding. One of the tools reviewed was the Iowa Infant Feeding Attitude Scale (IIFAS).^8^ The IIFAS was developed in the United States in 1999 by De la Mora et al. It has 17 items, scored using a 5-point Likert scale. It was tested in three phases, yielding Cronbach’s alpha values ranging from 0.68 to 0.86 and itemtotal correlation coefficients ranging from 0.070.23 to 0.45-0.69. The instrument is readily available and is widely used even in Malaysia. Other instruments have also demonstrated good reliability and validity in assessing infant feeding choices. However, relatively shorter and less-time consuming tools are more suitable for use in busy primary care clinics.

The IIFAS has demonstrated significant consistency and validity across multiple groups. Cronbach’s alpha coefficients ranging from 0.63 to 0.86 across diverse cultural adaptations demonstrate substantial reliability, with studies consistently indicating notable internal consistency.^9-11^ Factor analyses have demonstrated the construct validity for the scale, confirming its ability to accurately assess attitudes towards formula feeding and breastfeeding.^12,13^

The significant strength of the IIFAS lies in its adaptability across various linguistic and cultural contexts. The scale has been successfully translated and validated in Arabic, Polish, Spanish, Chinese and Ethiopian languages.^9,14^ This adaptability ensures its applicability in various settings, making it a versatile tool for public health initiatives and global research.

The IIFAS has also shown high precision in predicting nursing outcomes, including initiation and exclusivity.^15^ Moreover, a distinctive feature of the IIFAS is its capacity to assess both favourable and unfavourable perspectives on formula feeding and breastfeeding. This dual focus enables researchers to identify specific barriers to nursing and tailor interventions accordingly.^12,16^

The IIFAS is a concise 17-item questionnaire that is straightforward to administer and interpret. Research conducted in resource-limited settings has emphasised its simplicity.^14^ This practicality ensures that the instrument can be integrated into significant public health initiatives without imposing considerable burden on researchers or participants. The IIFAS has demonstrated its adaptability by being utilised across several groups, including pregnant women, postpartum mothers and fathers.^17^ It is distinctive in its focus on attitudes, a significant determinant of nursing behaviour, whereas other instruments primarily assess self-efficacy or nursing knowledge. For instance, the Breastfeeding Self-Efficacy Scale measures confidence in breastfeeding but does not assess attitudes towards breastfeeding or formula feeding.^18^ The Breastfeeding Knowledge Questionnaire prioritises knowledge over attitudes, making the IIFAS a more comprehensive tool for understanding the psychological factors influencing infant feeding choices.^14^

There are several reasons for validating a Malay version of the IIFAS. The successful adaptation and validation of the IIFAS across many cultural contexts, including Ethiopia and Indonesia, demonstrate its versatility and relevance to diverse populations.^19^ A Malay version would ensure an accurate evaluation of Malay mothers’ perspectives on breastfeeding by addressing their specific cultural and linguistic requirements.^20^ Higher scores signify a stronger inclination towards nursing, and the scale has been shown to correlate with the duration and exclusivity of nursing.^16^ Employing a validated instrument such as the IIFAS could assist Malaysia, where exclusive breastfeeding rates are below desired levels, in identifying barriers to breastfeeding, hence informing targeted interventions.^20^ Previous adaptations of the IIFAS have exhibited high reliability and validity, as evidenced by Cronbach’s alpha values indicating substantial internal consistency.^14,19^ Developing a Malay version necessitates equivalent rigorous validation processes to ensure its applicability within the Malaysian context.

## Methods

### Participant selection and study design

This validation study was conducted in Malaysia from March to June 2021 through an online survey. The source population was women with children under the age of 1 year. The inclusion criteria were the ability to understand the Malay language, age of more than 18 years and a youngest living child aged less than 1 year. The exclusion criteria were maternal illness or medications that prevent interactions with infants, infant transfer to the intensive care unit for more than 24 hours and infant condition that prevents effective feeding. Preliminary questions regarding age, age of the youngest child and previous illness were included in the online questionnaire to ensure compliance with the inclusion and exclusion criteria. Respondents who fulfilled the inclusion criteria were then permitted to proceed with completing the remainder of the questionnaire.

Snowball sampling was used in this study. Respondents were recruited through social media. An advertisement was posted in various social media platforms and groups of feeding mothers, with an incentive of honorarium. Participants were assured of the confidentiality of their data, and their informed consent to participate was obtained through a covering statement accompanying the questionnaire. The sample size was calculated using the Rule of Ten^20^ for factor analysis. The IIFAS has 17 items, and 170 participants were therefore needed. The sample size required was 204, considering a 20% non-response rate. For this study, the estimated sample size was 200. Breastfeeding is a sensitive topic; therefore, some mothers may hesitate to openly express their views and practices. This reluctance may lead to diminished response rates, especially in studies requiring extensive personal information.^21,22^ Some mothers may also decline to participate due to lack of awareness or time constraints.^23^ Larger sample sizes contribute to greater generalisability and accuracy of data.^19,24^ Thus, 522 questionnaires were distributed.

### Study instrument

The study instrument used in this study included items on the sociodemographic profile and background information of respondents and their infants, as well as the Malay version of the IIFAS. We evaluated the IIFAS for use in the Malaysian setting. Permission to adapt and use the original version of the IIFAS was obtained from the copyright holder, Arlene De la Mora. The IIFAS consists of 17 items, scored on a 5-point Likert scale ranging from 1 *(strongly disagree)* to 5 *(strongly agree).* The total IIFAS score ranges from 17 to 85. The score is then categorised into three groups. The first category is scored 70-85, which indicates positive attitudes towards breastfeeding. The second category is scored 49-69, which indicates neutral attitudes. The third category is scored 17-48, which indicates positive attitudes towards formula feeding.

### Instrument translation, face and content validation and administration

The IIFAS was translated using a forwardbackward process. Two independent translators conducted the forward translation. The translated versions were then reviewed and refined by a team consisting of two family medicine specialists and a public health physician, all proficient in both Malay and English, to produce the Malay version of the IIFAS. This version was subsequently sent to two professional translators for backward translation. The backward translations were compared with the original IIFAS by the research team. This comparison confirmed that the Malay version retained the meaning and content of the original version.

**Figure 1:**
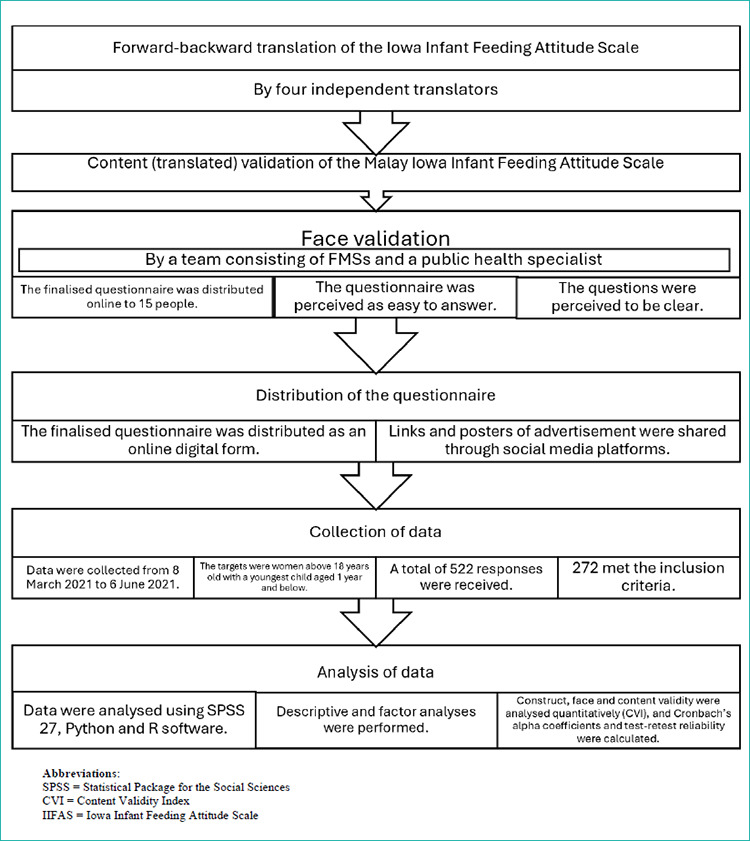
Flow chart of the study procedures.

The agreed Malay version was then subjected to face validation. It was distributed as an online questionnaire to 15 people to assess understanding and clarity. Minor amendments were made based on their feedback, with all respondents indicating that the questionnaire was easy to complete. Upon completion of the face validation, the finalised Malay version was approved, as shown in Figure 1. To ensure test-retest reliability, we conducted a pilot survey with a sample of 30 nursing women. The pilot test showed Cronbach’s alpha values exceeding the threshold of 0.7.

The questionnaire was digitised using Microsoft Forms. A link to the form was generated in both QR code and HTML formats. The Malay version of the IIFAS was then distributed online via social media platforms through the links, accompanied by advertisements and welcoming messages. The platforms used were Facebook and WhatsApp. The online form was open for responses from 8 March to 6 June 2021. The study procedures are shown in Figure 1.

### Data analysis

The data collected from the sociodemographic form and the Malay version of the IIFAS were entered into Microsoft Excel (Microsoft Corp., Redmond, WA, USA) and IBM SPSS Statistics for Windows, Version 27.0 (IBM Corp., Armonk, NY, USA) for analysis. Sociodemographic analysis was conducted by calculating the means and frequencies of the variables. Item descriptive analysis was first performed on the responses to the Malay version of the IIFAS. The reliability of the Malay version was measured through internal consistency, using Cronbach’s alpha coefficients, and test-retest reliability. The construct validity of the questionnaire was evaluated using Pearson correlation and principal component analyses. Additional statistical analyses, including parallel analysis and visualization, were conducted using Python (Python Software Foundation, Wilmington, DE, USA) and R software, Version 4.2.0 (R Foundation for Statistical Computing, Vienna, Austria).

## Results

During the period in which the online form was available, a total of 522 questionnaires were distributed. Two hundred seventy-two respondents who fulfilled the inclusion criteria were included in the analysis. All of these respondents also completed the retest of the questionnaire.

The sociodemographic profiles of the respondents are presented in the Table 1. The mean age of the 272 respondents was 30.1±4.2 years. Most (n=242, 89%) of them were Malay, while 261 (96%) were Muslim. A total of 270 (99.3%) respondents were married, as shown in Table 1. The mean number of living children was 1.9±2, and the youngest child’s mean Age was 5.3±5 months. All respnndents received 9ormal education, with 219 (80.5%) having post-secondary and tertiary educational levels and the remaining 53 (19.5%) having a secondary educational level. The majority (n=175, 64.3%) had a household income of less than RM 4970.00, with a mean income of RM 4734.17±3520.59. All respondents had no serious complications during and after pregnancy and delivery. Conversely, 175 (64.3%) gave birth through vaginal delivery, 74 (27.2%) through caesarean section and 23 (8.5%) through assisted vaginal delivery.

**Table 1:** Sociodemographic profiles of the respondents.

Characteristic	F	%
Age, year		
18-24	18	6.6
25-31	166	61.0
32-38	80	29.4
39-45	8	2.9
Ethnicity		
Malay	242	89.0
Others	30	11.0
Religion		
Islam	261	96.0
Others	11	4.0
Marital status		
Married	270	99.26
Divorced	2	0.74
Place of delivery		
Hospital	270	99.26
Clinic	2	0.74
TOTAL	272	100
Mode of delivery		
Forceps- or vacuum-assisted delivery	23	8.5
Caesarean section	74	27.2
Vaginal delivery	175	64.3
Level of education		
Secondary	53	19.5
Post-secondary and tertiary	219	80.5
Household income		
<RM 4970	175	64.3
RM 4971-10,970	87	32.0
>RM 10,971	10	3.7
Youngest child’s age, month		
0-2	74	27.2
3-5	67	24.6
6-8	76	27.9
9-12	55	20.2
No. of living children		
1	127	24.56
2	81	31.33
3	37	21.47
4	20	15.47
5	5	4.84
6	2	2.32

The descriptive analysis of the Malay version of the IIFAS is summarised in Table 2. Each item’s mean score and the percentage of responses on the five-point Likert scale are presented. Items 3, 12, and 13 showed the highest mean values, indicating strong agreement with breastfeeding-favourable statements, while items 4, 10, and 11 had the lowest means, reflecting disagreement with formula-feeding statements.

**Table 2:** Descriptive analysis of the questionnaire items.

Item	Mean	Likert scale response, n (%)				
		1	2	3	4	5
1	3.07	33 (12.1)	70 (25.7)	51 (18.8)	80 (29.4)	38 (14.0)
2	2.26	58 (21.3)	112 (41.2)	77 (28.3)	22 (8.1)	3 (1.1)
3	4.86	1 (0.4)	0	7 (2.6)	21 (7.7)	243 (89.3)
4	1.71	138 (50.7)	94 (34.6)	24 (8.8)	12 (4.4)	4 (1.5)
5	3.46	7 (2.6)	33 (12.1)	89 (32.7)	117 (43.0)	26 (9.6)
6	2.31	58 (21.3)	110 (40.4)	71 (26.1)	27 (9.9)	6 (2.2)
7	3.26	25 (9.2)	62 (22.8)	57 (21.0)	72 (26.5)	56 (20.6)
8	2.13	96 (35.3)	103 (37.9)	30 (11.0)	28 (10.3)	15 (5.5)
9	3.62	9 (3.3)	40 (14.7)	68 (25.0)	84 (30.9)	71 (26.1)
10	2.23	50 (18.4)	137 (50.45)	63 (23.2)	16 (5.9)	6 (2.2)
11	1.81	105 (38.6)	129 (47.4)	23 (8.5)	14 (5.1)	1 (0.4)
12	4.69	0	2 (0.7)	6 (2.2)	67 (24.6)	197 (72.4)
13	4.76	1 (0.4)	0	10 (3.7)	42 (15.4)	219 (80.5)
14	2.24	50 (18.4)	129 (47.4)	72 (26.5)	19 (7.0)	2 (0.7)
15	4.09	1 (0.4)	12 (4.4)	58 (21.3)	91 (33.5)	110 (40.4)
16	4.44	4 (1.5)	8 (2.9)	23 (8.5)	65 (23.9)	172 (63.2)
17	4.03	11 (4.0)	23 (8.5)	46 (16.9)	58 (21.3)	134 (49.3)

**Figure 2:**
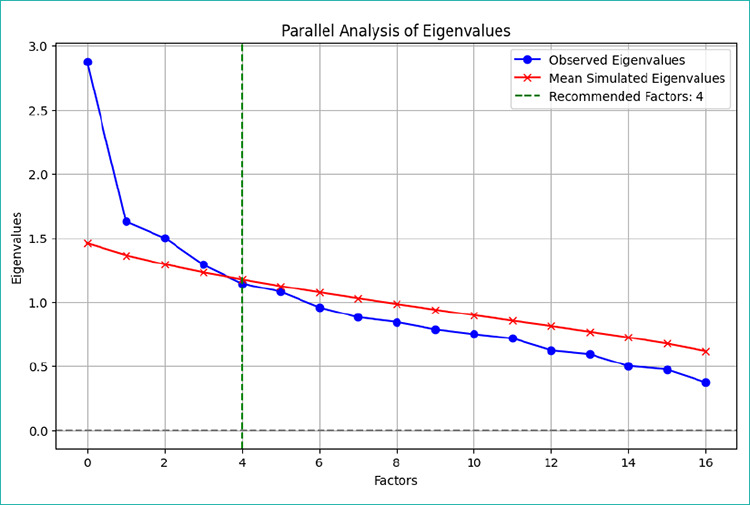
Parallel analysis of eigenvalues.

The psychometric evaluation of the IIFAS-M demonstrated satisfactory validity and reliability.Table 3 summarises the main findings. The Kaiser-Meyer-Olkin (KMO) value was 0.857, indicating excellent sampling adequacy, and Bartlett’s test of sphericity was significant (χ^2^ = 613.70, p < 0.001), confirming the data’s suitability for factor analysis. The overall Cronbach’s α was 0.832, reflecting good internal consistency. Corrected item-total correlation (CITC) values ranged from -0.060 to 0.172, with items 6, 7, and 17 showing the strongest correlations with total scores. Normality plots confirmed that data were not normally distributed, and most items demonstrated moderate inter-item correlations (r = 0.3-0.5). Four factors were extracted based on the parallel analysis of eigenvalues, accounting for approximately 43% of total variance (Factor 1 = 16.9%, Factor 2 = 9.6%, Factor 3 = 8.8%, and Factor 4 = 7.6%). These results are illustrated in Figure 2.

**Table 3:** Validity and reliability test results.

Item	KMO value	Item	KMO value	Item	KMO value
Q1	0.7887	Q9	0.7934	Q17	0.8850
Q2	0.8150	Q10	0.7917		
Q3	0.8540	Q11	0.7085		
Q4	0.8302	Q12	0.8691		
Q5	0.8132	Q13	0.8252		
Q6	0.8643	Q14	0.7110		
Q7	0.8461	Q15	0.8439		
Q8	0.7602	Q16	0.7990		

KMO value: 0.8570225 Bartlett’s test of sphericity: χ^2^ = 613.6975, P=0.0000 Cronbach’s alpha value=0.8323 KMO: Kaiser-Meyer-Olkin

## Discussion

The analysis revealed high mean scores for certain items, particularly items3 and 13, which had mean scores of 4.86 and 4.76, respectively. Notably, 89.3% and 80.5% of the respondents strongly agreed with these items. This trend mirrors findings from a Greek study, where the mean scores were 4.8 for item 3 and 4.4 for item 13.^25^ Similarly, the (Chinese version yielded mean scores of 4.74 for item 3 and 4.62 for item 13.26 while a Canadian validation stuUo found mean scores of 4.4 Uon item 3 and 4.02 Oor item 13.27. This suggests that mothecs are generally aware of the benefits ofbreastfeeding.

Conversely, the lowest mean scores were noted in items 4 and 11, which scored 1.71 and 1.81, respectively; approximately 85.3% and 86% of the respondents either disagreed or strongly disagreed with these items. These low scores contrast with those in other validation studies: The Canadian study reported mean scores of 3.68 for item 4 and 4.31 for item 11,^27^ and the Chinese version yielded mean scores of 3.82 for item 4 and 4.11 for item 11.^26^ This discrepancy may reflect a better understanding of breastmilk nutrition and the supportive environment for mothers in Malaysia compared to other countries.

The distribution of the scores deviated from normality, differing from the findings of Mora et al.^7^ A new cut-off value for the Malay version of the IIFAS was established to better represent attitudes towards infant feeding. The original IIFAS scoring for positive attitudes towards formula feeding was 17-48, while the Malay version used a scoring ranging from 17 to 59. The original scoring for neutral attitudes was 49-69, whereas the Malay version set the scoring at 60-70. Finally, the original scoring for positive attitudes towards breastfeeding ranged from 70 to 85, while the Malay version utilised a scoring that ranged from 71 to 85. These differences may reflect an enhanced understanding of breastfeeding compared to that during the original IIFAS development.

To assess whether the Malay version of the IIFAS accurately measures attitudes towards infant feeding, we analysed the validity of the items. The Pearson correlation analysis indicated that all items were significant, with P-values less than 0.05, confirming their validity.^28^

The principal component analysis began with an examination of the correlation matrix, where values should ideally exceed 0.3 for at least one item. In our study, a few items (items 1, 10 and 17) fell below this threshold, warranting further observation.^29^

The KMO measure of sampling adequacy was 0.857, with a Bartlett’s test of sphericity significance value of less than 0.001, indicating suitability for principal component analysis. The analysis of the eigenvalues and screen plots revealed six extracted components. The rotated component matrix, using Varimax rotation with Kaiser normalisation, showed factor loadings of 0.4 or higher, with each component containing a minimum of two items. Consequently, all items were retained, contrasting with other validity studies of the IIFAS or its translated versions, which necessitated item removal, such as studies conducted in Singapore and Japan.9,^30^ In those studies, items 5 and 17 were discarded due to low factor loadings and commonalities.

The number of extracted components aligns with the original IIFAS development, where items were categorised into 10 topic areas related to breastfeeding and formula feeding processes and products. This finding is consistent with that of an Ethiopian study that also extracted six components with eigenvalues exceeding 1.^13^

The first reliability assessment involved calculating the internal consistency values using Cronbach’s alpha. The overall Malay version reliability value before components adjustment for all 17 items was 0.657, which is lower than that of the original IIFAS, reported at 0.855. A comparable reliability value was reported in a Japanese study (0.66).^31^ According to the literature, a Cronbach’s alpha value of 0.4—0.6 is deemed acceptable for indicating reliability.^32^

The internal consistency of the items within the Malay version of the IIFAS ranged from 0.2 to 0.6, with the lowest value stemming from the sixth component, which included items 1 and 17. However, deleting these items would reduce the overall Cronbach’s alpha value below 0.657. Thus, these items were retained due to their factor loading exceeding 0.4. This situation differs from the Japanese study, where items had negative factor loadings and low internal consistency values.^31^ A low Cronbach’s alpha value may result from a limited number of questions, poor interitem correlations or heterogeneous constructs. Therefore, supplementary tests to assess reliability are recommended.^33^

The second reliability assessment was conducted through a test-retest method. For the Malay version of the IIFAS, the Pearson correlation coefficient was 0.8, with a P-value of less than 0.001. This finding aligns with the test-retest reliability result obtained in a Singaporean study (0.85).^9^ Other validation studies did not report test-retest reliability.^12,29,34,35^

The differences in the item loadings between the Malay version and other versions of the IIFAS may be attributed to demographic, cultural and methodological factors that warrant further investigation. For instance, differences in racial backgrounds between populations in Malaysia and Iowa may affect the responses to certain items, especially those relating to alcohol consumption. This is because Muslims are prohibited from consuming alcohol. Moreover, contextual factors such as educational level and income may contribute to variations in responses.^19^ Aspects of the face validation and the wording and rewording of questions may also present further areas for exploration.

### Study limitations

The questionnaire was distributed through online advertising, allowing any Malaysian to participate. This method presents a limitation, as respondents may have encountered questions or uncertainties while completing the questionnaire that could not be addressed in real time. Additionally, the variations in the response rates across different online platforms may have influenced the study results, potentially introducing bias into the data collected. Alternative data collection methods, such as face-to-face interviews, could yield different results and offer deeper insights. However, during the study period, the COVID-19 pandemic imposed significant constraints on face-to-face interactions, and factors such as time limitations and mobile data availability further hindered the ability to conduct online interviews with mothers. Future research could benefit from expanding the study population to include a more diverse demographic, which may provide a broader perspective on attitudes towards infant feeding across various cultural and socioeconomic contexts.

## Conclusion

This study validated the Malay version of the IIFAS. Mothers were contacted through social media platforms and participated in a survey. The factor analysis identified four significant factors based on the eigenvalues, indicating meaningful relationships within the dataset. The strong CITC values for q6, q7 and q17 suggest their importance in measuring attitudes. The correlation matrix revealed significant relationships among the variables, with a KMO measure of 0.8570, indicating excellent sampling adequacy. Bartlett’s test confirmed the dataset’s suitability for analysis (P<0.0001), and the Cronbach’s alpha value of 0.8323 demonstrated good internal consistency of the scale. The Malay version of the IIFAS is a validated and reliable tool for assessing attitudes towards infant feeding among the Malayspeaking population. The study underscores the influence of cultural and socioeconomic factors on these attitudes and suggests potential pathways for further research to enhance the instrument’s applicability across diverse populations.

## Data sharing statement

Data are available upon request.

- The validated questionnaire can be used to assess attitudes towards infant feeding choices among women in the local context.
- The Malay version of the Iowa Infant Feeding Attitude Scale is a reliable tool that can be used in future research in promoting and supporting breastfeeding as the optimum feeding method for infants.
